# Drug screening of biopsy-derived spheroids using a self-generated microfluidic concentration gradient

**DOI:** 10.1038/s41598-018-33055-0

**Published:** 2018-10-02

**Authors:** Theresa Mulholland, Milly McAllister, Samantha Patek, David Flint, Mark Underwood, Alexander Sim, Joanne Edwards, Michele Zagnoni

**Affiliations:** 1 Centre for Microsystems and Photonics, Electronic and Electrical Engineering, University of Strathclyde, Glasgow, G1 1XW UK; 2 Institute of Cancer Science, College of Medical, Veterinary and Life Sciences, University of Glasgow, Glasgow, G61 1QH UK; 3 Strathclyde Institute of Pharmacy and Biomedical Sciences, University of Strathclyde, Glasgow, G4 0RE UK; 4 Department of Urology, Queen Elizabeth University Hospital, Glasgow, G51 4TF UK; 5 AMS Biotechnology (Europe) Ltd, Milton Park, Abingdon, OX14 4SE UK

## Abstract

Performing drug screening of tissue derived from cancer patient biopsies using physiologically relevant 3D tumour models presents challenges due to the limited amount of available cell material. Here, we present a microfluidic platform that enables drug screening of cancer cell-enriched multicellular spheroids derived from tumour biopsies, allowing extensive anticancer compound screening prior to treatment. This technology was validated using cell lines and then used to screen primary human prostate cancer cells, grown in 3D as a heterogeneous culture from biopsy-derived tissue. The technology enabled the formation of repeatable drug concentration gradients across an array of spheroids without external fluid actuation, delivering simultaneously a range of drug concentrations to multiple sized spheroids, as well as replicates for each concentration. As proof-of-concept screening, spheroids were generated from two patient biopsies and a panel of standard-of-care compounds for prostate cancer were tested. Brightfield and fluorescence images were analysed to provide readouts of spheroid growth and health, as well as drug efficacy over time. Overall, this technology could prove a useful tool for personalised medicine and future drug development, with the potential to provide cost- and time-reduction in the healthcare delivery.

## Introduction

Over the last 10 years, the use of three-dimensional (3D) cell culture models and consideration of the extracellular microenvironment have been shown to be of immense importance when studying cancer therapies and the mechanisms leading to drug resistance and metastasis^1–3^. Cell signalling, cell-to-cell contact, chemical and gas concentration gradients are altered in two-dimensional (2D) cultures with respect to the 3D *in vivo* microenvironment^4^. These differences can affect mechanistic studies, but especially drug screening, where cells can be more or less sensitive in 3D than in 2D, or even completely resistant^5^, depending on the drug’s mechanism of action^6,7^. Furthermore, 2D culture neglects the presence of varying degrees of cellular metabolic activity, typical of *in vivo* tumours^3,4^. Recently, the drive to create more refined *in vitro* 3D tumour models, which mimic the heterogeneous human tumour microenvironment, has resulted in the use of human tumour tissue for the generation of multicellular spheroids^8^. Animal-based methods, such as patient-derived xenografts (PDX) and PDX-derived spheroids, offer increased physiological relevance and are superior to *in vitro* models for the study of malignant transformation, invasion and metastasis. However, the compromised immune system of the host mice and the lack of human stromal tissue are reasons for their poor predictive value^9–11^. Conventional methods used for the generation spheroids, such as spinner flask, forced-floating and hanging drop techniques^4,12,13^ offer the opportunity for high-throughput screening. Other advanced techniques utilise scaffolds, magnetically levitate spheroids or use aqueous biphasic microtechnology for the generation and culture of spheroids^14,15^. However, these methods have the disadvantage of requiring relatively large volumes of reagents and cellular material^4^, which is a particular challenge when working with biopsy-derived tumour tissue^8^.

Microfluidic technologies have increasingly been used for spheroid-based assays and offer viable solutions when working with biopsy-derived tumour tissue, providing precise control over the cellular microenvironment, 3D cell culture and medium- to high-throughput readouts in a cost-effective manner^16–19^. Importantly, laminar flow conditions enable the formation of compound concentration gradients, leading to multiple experimental conditions to be tested simultaneously within a micro-scale device. A variety of designs have been proposed, relying on T-junction gradient generators and tree-like microfluidic structures, which have been adapted for chemotaxis investigation and drug testing^20,21^. However, a common disadvantage of all these approaches is that they typically require external instrumentation for fluid actuation, such as syringe pumps or pressure controllers, and multiple tubes to be connected to the device. These can be bulky, expensive and hinder the ease of use of microfluidic technology in research and industrial environments. Alternative approaches have been proposed to generate stable concentration gradients without the use of external equipment^22^, but until now this has not been applied to spheroid-based drug screening.

Here, we present a microfluidic platform and associated cell culture protocols, aimed to facilitate extensive screening of cancer cell-enriched multicellular spheroids derived from human tumour biopsies. Novel aspects of our microfluidic design include self-generating perfusion of nutrients and repeatable, long-lasting drug concentration gradients across an array of hundreds of spheroids without requiring equipment to be connected to the device for fluid actuation. Using this system, several concentration response curves (8-point curves obtained from a starting sample of ~50,000 cells, with each point averaging 10–24 spheroids) were obtained per biopsy, providing information on drug efficacy over time from the analysis of brightfield and fluorescence images.

Overall, this is the first example of an equipment-free, lab-on-a-chip platform enabling miniaturised compound screening on 3D tumour models from biopsy tissue with a throughput that is 100-fold greater than any previously described for spheroids overall^8,23–26^. This novel approach offers an animal-free, practical solution to personalised drug screening of 3D tumour biopsy preparations.

## Methods

### Device design and fabrication

**Figure 1:**
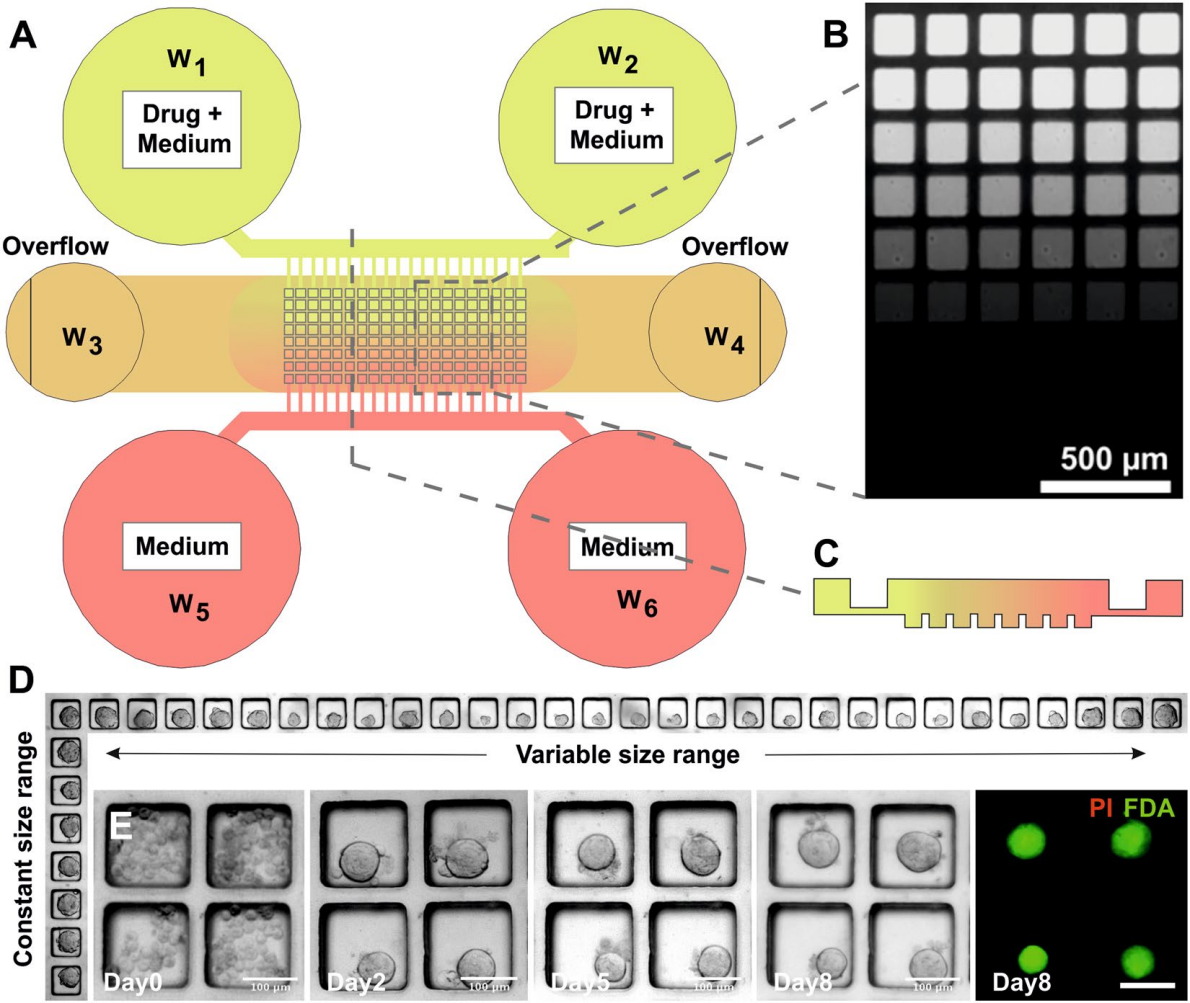
Microfluidic device layout and functions. (**A**) Schematic representation of the device structure, comprising a microchannel network and an array of square micro-wells, with a width of 150 µm and a depth of 180 µm. By seeding a single cell aggregate into the central channel, compact spheroids were formed over 48 hours. The microfluidic network enabled both to perfuse fresh medium, and to create a compound concentration gradient across the array. (**B**) Fluorescent image showing a calcein concentration gradient over the micro-well array. (**C**) Schematic cross-section of the gradient generated along a column of the spheroid array. (**D**) Representative images of spheroid size distribution obtained along rows and columns of the micro-well array. (**E**) Example of prostate tumour biopsy-derived spheroids formed from a single cell suspension and subsequent culture in the micro-wells (vehicle control; brightfield images from day 0 to day 8; fluorescent image from viability stain on day8 using FDA (green) and PI (red)). Scale bars = 100 µm.

Multi-layered microfluidic devices were fabricated using standard soft- and photo-lithography techniques and consisted of two polydimethylsiloxane (PDMS) parts bonded together (Fig. 1). Briefly, both PDMS parts were replica moulds from photoresist-patterned silicon wafers. The wafers were fabricated by using SU-8-3035 and SU-8-3010 (3000 Series, MicroChem Corp.), according to the manufacturer’s protocol. Spin-coated resist was exposed to collimated UV light through a photomask (JD Photo-Tools, UK) and developed using MicroPosit EC solvent (Rohm and Haas, US). In order to prevent the adhesion of PDMS to the patterned silicon wafer, 1 H,1 H,2 H,2H-perfluorooctyl-trichlorosilane (Sigma Aldrich, UK) was applied by vapour deposition (45 minutes) to each wafer. To fabricate devices, PDMS prepolymer (Sylgard 184, Dow Corning) was mixed with curing agent in a 10:1 ratio, poured on the patterned wafers and cured at 85 °C for at least 2 hours. To create an approximately flat and uniformly thick (~1 mm) bottom PDMS layer, plastic spacers and a glass slide were coated with 0.1% hydroxymethylcellulose (HPMC, Sigma) to prevent PDMS adhesion. The spacers were placed on the wafer prior to pouring PDMS, then the glass slide was placed on top of the spacers and secured to the wafers using metal clamps. Once cured, PDMS devices were peeled from the moulds, cut to size and open wells were created using a surgical biopsy punch (4 or 8 mm diameter, Miltex). The PDMS layers were then cleaned and permanently bonded together using oxygen plasma surface treatment (Pico plasma cleaner, Diener electronic). Bonded devices were then baked at 85 °C for at least 30 minutes and stored dry. Prior to cell seeding, the devices were exposed to oxygen plasma for 2 minutes and a surfactant solution of 1% Synperonic F108 (Sigma-Aldrich, UK) in deionised water was immediately pipetted into the device to render the PDMS inner channel surfaces non-adherent and biocompatible. The devices were incubated at 37 °C and 5% CO_2_ for 24 hours. The remaining surfactant solution was then removed by washing the device with phosphate buffered saline (Fisher Scientific), followed by injection of incomplete medium.

### Cell culture

Human high-grade glioma cells (UVW) were provided by Dr Marie Boyd (University of Strathclyde). UVW cells and spheroids were maintained at 37 °C and 5% CO_2_ in minimum essential medium (Gibco), supplemented with 10% fetal bovine serum (Gibco), 2.5 μg ml^−1^ Fungizone (Gibco), 100 U ml^−1^ Penicillin/Streptomycin (Gibco) and 2 mmol l^−1^ L-Glutamine (Gibco). LNCaP cells were provided by Prof. Hing Leung (University of Glasgow) and were maintained at 37 °C and 5% CO_2_ in RPMI-1640 supplemented with 10% fetal bovine serum (Gibco), 2.5 μg ml^−1^ Fungizone (Gibco), 100 U ml^−1^ Penicillin/Streptomycin (Gibco), 1 mmol l-1 Sodium pyruvate (ThermoFisher) and 2 mmol l^−1^ L-Glutamine (Gibco).

Human prostate samples were obtained from patients undergoing transrectal ultrasound (TRUS) biopsy for investigation of prostate cancer and cultured using a simple, previously optimised, 2D culture method. Samples were stored in a serum-free RPMI (Invitrogen, UK) at 4 °C overnight. Biopsies were then minced and suspended in serum-free RPMI and incubated at 37 °C in 5% CO_2_ overnight. A cell strainer was used to remove any single cells, including fibroblasts, from the tissue pieces. The tissue pieces were washed three times in 10 ml of PBS (Invitrogen, UK). The remaining tissue pieces were re-suspended in 5 ml of primary prostate cell media, containing a range of supplements to enhance epithelial growth and reduce fibroblast populations (Table S1 in SI). The tissue pieced were cultured in a T-25 Matrigel-coated flask at 37 °C in 5% CO_2_, left undisturbed for 7 days to allow attachment of the cells/tissue pieces to the flask. Media was renewed after 7 days, and every 2–3 days thereafter. Cells were passaged at approximately 70% confluency to maintain a monolayer and prevent overcrowding. Cells were then washed twice in PBS heated to 37 °C to remove any traces of media. Cells were incubated in 3 ml of trypsin (Invitrogen, UK) for 5 minutes in 5% CO_2_ at 37 °C. Once cells were detached from the flask, they were washed three times in PBS. Finally, the cells were re-suspended in 10 ml of primary prostate cell media and cultured in a T-75 flask at 37 °C in 5% CO_2_. Again, cells were cultured to approximately 70% confluency before being passaged 1:4 into four T75 flasks. Prior to microfluidic experiments, cells were detached from the bottom of T75 culture flasks (Corning) using 0.25% Trypsin-EDTA (Gibco), dead cells excluded using Trypan Blue (Gibco) and cell suspensions were prepared in complete medium. One flask was kept for spheroid formation in microfluidic devices and remaining flasks were used for characterisation by RT-qPCR.

### Ethical approval and informed consent

All methods involving the use of human tissue samples were carried out in accordance with relevant guidelines and regulations. All relevant regulatory approvals required for the use of anonymised human tissue and experimental protocols to conduct this work was obtained by the West of Scotland Research Ethics Service (ref. 16/WS/0015). Informed consent was obtained from all subjects.

### RT-qPCR

RNA was extracted from biopsy preparations and cDNA synthesis was performed as detailed in SI. Quantitative reverse transcription polymerase chain reaction (RT-qPCR) was performed to allow comparison between gene expression in the control sample (PNT2 benign cell line) and the immortalised prostate cell lines and primary prostate cells. A 96 well optical fast PCR plate was used. The following quantities were added in each well: 40 ng cDNA from the cell line of interest, 10 μL of master mix (Life Technologies), 5 μL nuclease-free water and 1 μL of gene expression assay. The gene expression assays used in this study were all Taqman Gene Expression Assays and included predesigned primers and probes sets for the androgen receptor (AR), fatty acid synthase (FASN), kallikrein-3 (KLK-3 gene for the prostate specific antigen protein), golgi membrane protein 1 (GOLM1) and alpha-methylacyl-CoA racemase (AMACR) (Applied Biosystems, cat. no. Hs00171172_m1, Hs01005622_m1, Hs02576345_m1, Hs00213061_m1, and Hs01091292, respectively). Blank control wells containing only the mixture and no cDNA were included in each plate to exclude contamination. Plates were sealed and centrifuged at 1200 rpm for 3 minutes. Air bubbles were removed using a Microlance needle. RT-qPCR was performed using an ABI 7500 real time PCR machine (Applied Biosystems). Samples were heated at 50 °C for two minutes, 95 °C for 10 minutes then 40 cycles of 95 °C for 15 seconds and 60 °C for one minute. Gene expression was normalised to the beta-actin (ActB) housekeeping gene (Applied Biosystems, cat. no. Hs01060665_g1). The comparative cycle threshold (ΔΔCt) method was used to quantify relative gene expression.

### Spheroid formation

Cells were seeded into the microfluidic devices as a single cell suspension. For UVW and LNCaP cells, 12 µL of cell suspension at a concentration of 7 × 10^6^ cells/mL were pipetted into the inlet open well of the culture channel and sedimented at the bottom of the microwells of the spheroid array. For primary prostate cells, a cell suspension of 2 × 10^6^ cells/mL was prepared and 12 µL were seeded into each device. Excess cells in the outlet wells were washed out. Due to the non-adherent condition of the inner channel surfaces, cells typically aggregated into compact multicellular spheroids within 48 hours. Medium was exchanged every 24 to 48 hours, depending on the cell type used, using a micropipette.

### Viability staining

Spheroid viability was assessed by using propidium iodide (PI, Sigma-Aldrich) at 20 µg mL^−1^, fluorescein diacetate (FDA, Sigma-Aldrich) at 8 µg mL^−1^ and Hoechst33324 at 5 µmol L^−1^ (Thermo Scientific). Spheroids were incubated with the dyes in the microfluidics for 15 minutes at room temperature. Excess dye was then washed off using PBS for 5 minutes and spheroids were imaged immediately. After staining, experiments were terminated.

### Microfluidic drug screening

A 1.6 mmol L^−1^ stock solution of cisplatin (Sigma-Aldrich) was produced by dissolving cisplatin in 0.9% NaCl solution (Sigma-Aldrich) and stored at 4 °C for up to 30 days. A 10 mmol L^−1^ stock solution of Docetaxel (Selleckchem) and a 10 mmol L^−1^ solution of Enzalutamide in DMSO (Selleckchem) was stored at −20 °C. All working drug solutions were prepared in complete medium and used immediately.

Spheroids were formed and initially cultured in the microfluidics for 3–5 days. Subsequently, all medium was removed from the devices and replaced by either a drug solution in single channel devices (for comparison against results from drug gradient generating devices) or by both fresh medium and a drug solution in the gradient generating devices. Drugs were left to incubate in the devices for 12 hours at 37 °C and 5% CO_2_. Drug solutions were then washed out using complete medium and spheroid responses were monitored for at least 3 days (and up to 14 days) post drug exposure using brightfield and epifluorescence microscopy. Control experiments were performed for each cell line and biopsy culture and for different set of experiments. Concentration ranges for each drug were obtained from the literature, when available for 3D models, or modified from 2D derived data.

### Calcein gradient generation

To characterise the formation of a microfluidic compound concentration gradient, calcein was used to image the temporal evolution of the gradient via epifluorescence microscopy. Calcein (Sigma-Aldrich) was dissolved in deionised water to produce a 100 µM solution and used in place of a drug, following the developed protocols. Fluorescent images were acquired using an inverted microscope (Axiovert A1, Zeiss) and a CMOS camera (Orca Flash 4.0, Hamamatsu) over 16 hours. Devices were kept in high humidity conditions inside a microscope stage incubator (Tokai Hit INUB-WELS-F1, Japan) during time-lapse recording.

### Numerical simulation

**Figure 2:**
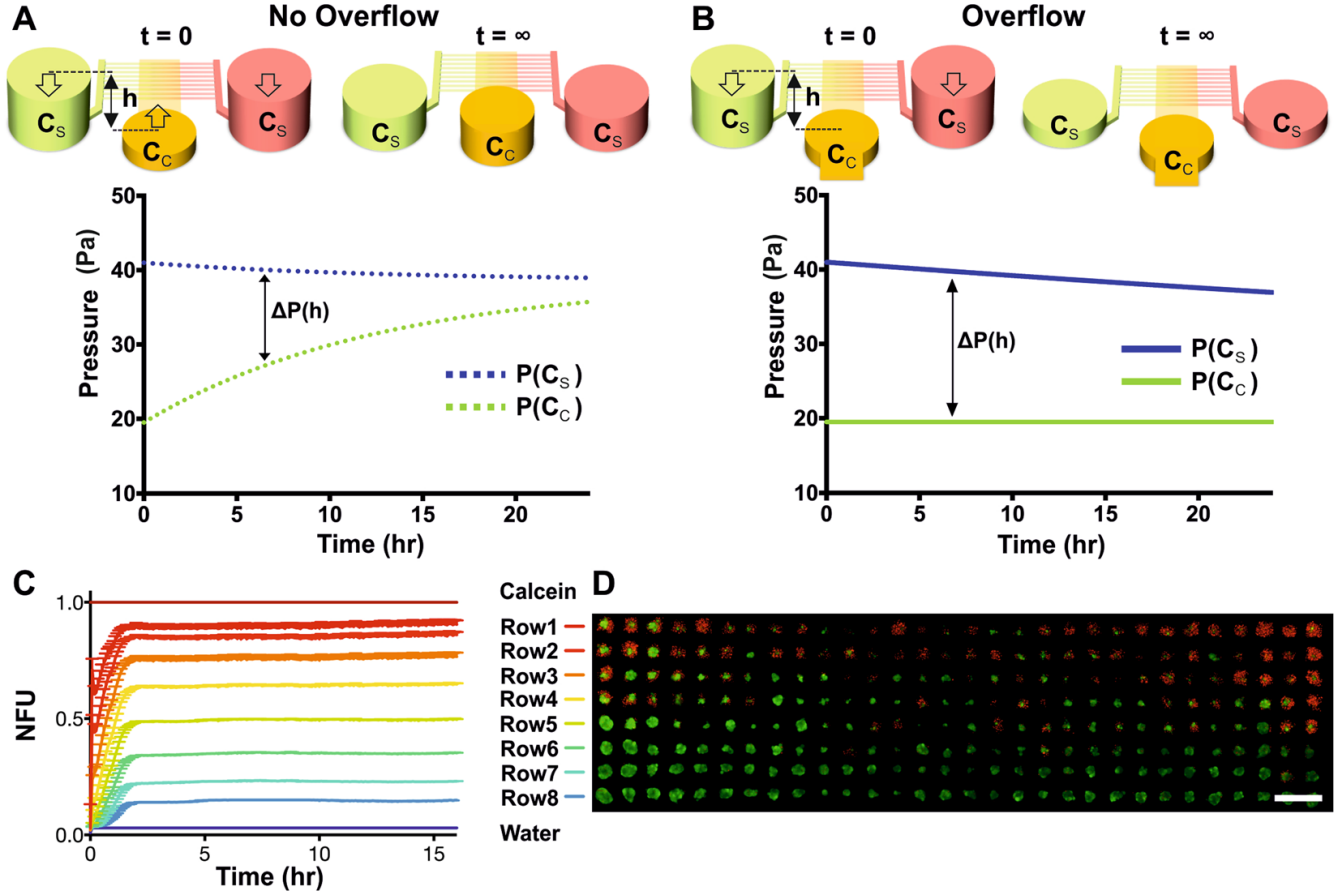
Overflow ports enabled the formation of long-lasting chemical concentration gradients without external fluid flow actuation. (**A**,**B**) Schematic illustration and temporal evolution of the hydrostatic pressure difference ΔP(h) for the same microfluidic device in the absence (**A**) and presence (**B**) of overflow ports. (**C**) Plot of the mean fluorescent intensity of all the wells in a row of the spheroid array obtained using calcein (100 µM) to visualize the formation of a stable concentration gradient lasting for over 15 hours (Video S2 in SI). Error bars represent standard error of the mean and show the variability between wells in each row. (**D**) Overlay epifluorescence microscopy image of live/dead staining (green = FDA, red = PI) of UVW glioma spheroids following a cisplatin concentration gradient for 12 hours. Scale bar is 500 µm.

To estimate the temporal evolution of the hydrostatic pressure values within each open well of the device during compound concentration gradient formation, numerical and analytical models of the fluid behaviour in the device were developed. First, an equivalent electrical circuit of the microfluidic device structure was created using Orcad PSpice, where a distributed resistive network represented the microfluidic channel network and capacitors represented the open well reservoirs (Fig. S1 in SI). The initial conditions (amount of electrical charge in each capacitor) were set to represent the starting volume of fluids in each well at the start of the experiment. Values of equivalent electrical resistance and capacitance were calculated as previously described^27,28^. Following this, an analytical expression of the hydrostatic pressure was derived using a simplified equivalent electric circuit (Fig. S2 in SI) and used as an input for finite element model (FEM) simulation. To estimate the temporal evolution of microfluidic compound concentration gradients, a 3D FEM model of the microfluidic device was built using COMSOL 3.5 (Fig. S3A,B in SI). The Navier–Stokes equations were solved to model pressure-driven fluid transport alongside Fick’s law equations to model the compound diffusive transport. Diffusion coefficients were obtained from the literature when possible or estimated from the compound molecular weight and adjusted according to experimental conditions. The microfluidic concentration gradient obtained from the simulation (Fig. S3C in SI) was compared against experimental results using calcein (Fig. 2C) showing good accuracy of the numerical model (Fig. S3D). Simulations for all compounds tested were carried out to estimate variation in the compound concentration gradient due to different diffusion coefficients (estimated in a range 0.2–0.8 × 10^−10^ m^2^ s^−1^).

### Microscopy and image analysis

Spheroids were monitored every 24–48 hours using an inverted microscope (Observer A1, Zeiss) connected to an Orca Flash 4.0 camera (Hamamatsu). For long-term live-cell imaging, a microscope stage incubator (Tokai Hit INUB-WELS-F1, Japan) was used to maintain high humidity condition whilst keeping the device at 37 °C and 5% CO_2_. Images were analysed and data processed using ZEN Blue, Fiji^29^ and Matlab R2014b. In-house developed Matlab routines were used to extract spheroid area and perimeter from the brightfield images, as well as spheroid areas and dye intensity from the fluorescent images.

### Screening assay readouts

**Figure 3:**
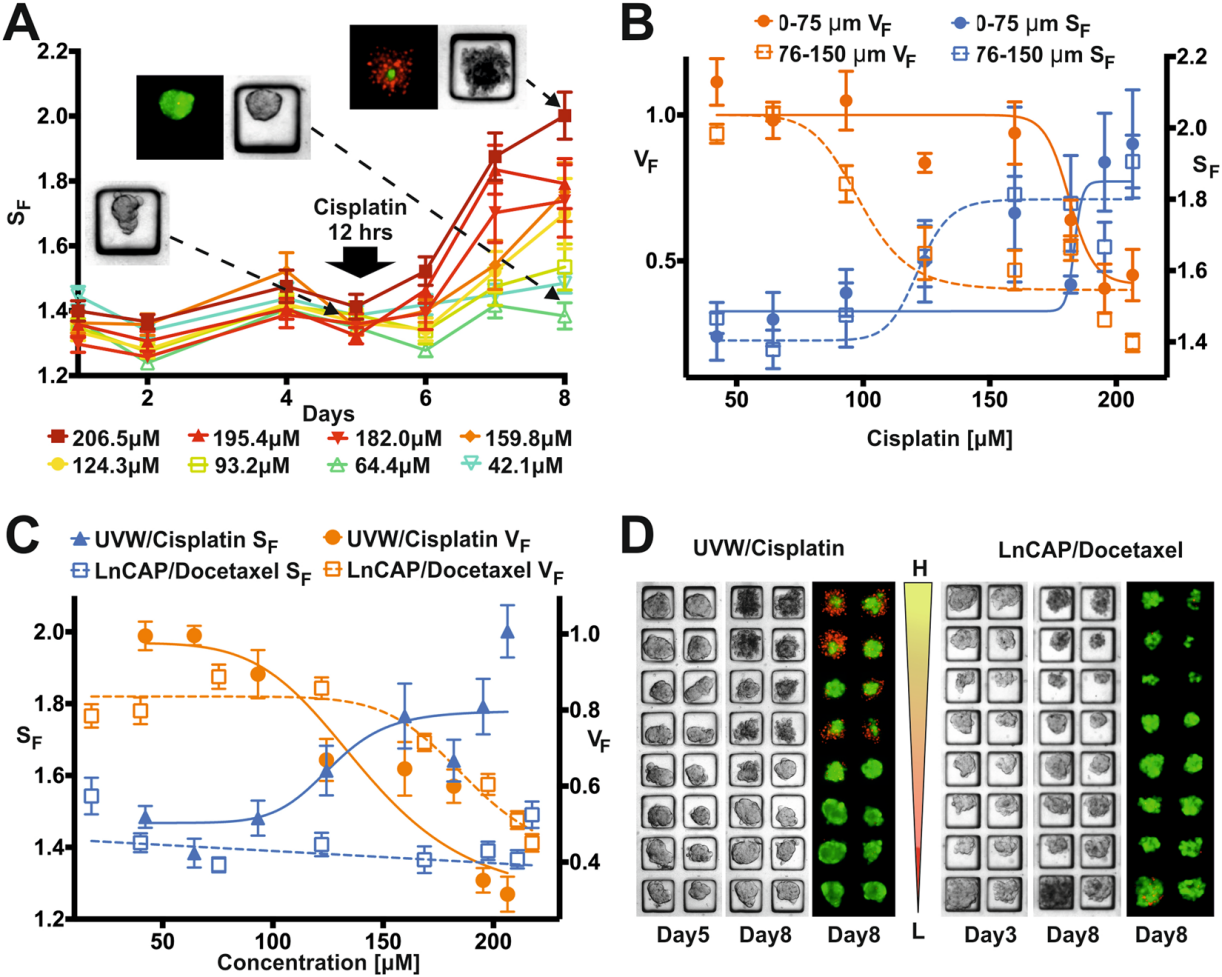
Screening readouts. (**A**) Temporal evolution of the average shape factor, S_F_, in each row of the spheroid array when using UVW cells. Each row was exposed to a decreasing concentration of cisplatin for 12 hours (as estimated from the numerical simulation), from 206.5 µM (Row 1) to 42.2 µM (Row 8). Inserts show brightfield and fluorescent images of representative spheroids prior to drug application (day 5) and after viability staining (day 8, red = PI, green = FDA) for different positions in the array. (**B**) 8-point concentration response curves obtained from data analysis averaging outcomes from each row of the spheroid array using UVW and cisplatin (n = 24). Both viable fraction, V_F_, and shape factor, S_F_, are plotted for the two spheroid size groups analysed: Group 1 = 0–75 µm and Group 2 = 76–150 µm. EC_50 VF Group 1_ = 181.5 µM, EC_50 VF Group2_ = 98.9 µM, EC_50 SF Group1_ = 183.5 µM, EC_50 SF Group2_ = 121.8 µM. (**C**) Comparison between 8-point concentration response curves obtained by exposing UVW spheroids to cisplatin and LNCaP spheroids to docetaxel in gradient devices for 12 hours. Both viable fraction, V_F_, and shape factor, S_F_, curves are plotted for all the spheroids in the array. Values are shown as mean (of n = 24 spheroids) ± standard error of the mean. (**D**) Representative brightfield and fluorescent images obtained from UVW-cisplatin (day 5 and 8) and LNCaP-docetaxel experiments in gradient devices (day 3 and 8), confirming successful gradient formation and consequent drug effects.

In addition to providing semi-quantitative information of drug efficacy using live-dead staining (Fig. 2D), the brightfield image of each spheroid was processed to estimate its health using a shape factor parameter, *S*_*F*_, described by the following equation:

1 $${S}_{F}=\frac{{P}^{2}}{4\pi A},$$

where *P* is the perimeter (*P* = 2*πr*) and *A* (*A* = *πr*^2^) is the area of the spheroid, respectively. Whilst healthy spheroids maintained a smooth perimeter and a spherical outline (R ~ 1), unhealthy spheroids showed sign of disaggregation and a rougher outline (R > 1) that was directly proportional to the drug concentration used. Such a level of disaggregation was quantified utilising equation (1)^13,30,31^ (Fig. 3B). Additionally, as a measure of drug efficacy, the viable fraction, *V*_*F*_, was calculated by processing fluorescent images in combination with brightfield images using equation (2):

2 $${V}_{F}=\frac{Are{a}_{FDA}}{Are{a}_{BF\_PtD}},$$

where *Area*_*FDA*_ is the area of the spheroid extracted from the fluorescent image from the FDA staining, representing the area of the spheroid that is assumed viable. *Area*_*BF_PtD*_ is the area of the spheroid, prior to drug incubation, extracted from the brightfield image. Values of V_F_ ≥ 1 indicate a spheroid that has either grown over time or has remained unaffected by the treatment with respect to its healthy state prior to drug incubation, whilst V_F_ < 1 indicates a detrimental effect of the drug or an unhealthy spheroid.

### Statistical analysis

Graphpad Prism 7 was used for plotting data and for statistical analysis. All data is presented as mean ± standard error of the mean, using bar graphs or scatter plots with sigmoidal fitting. Results were compared using two-way ANOVA tests, with differences considered significant when P < 0.05. The Pearson correlation coefficient was calculated using Graphpad Prism 7.

## Results

### Device structure and spheroid culture

The microfluidic device structure is composed of two layers: a bottom layer (~1 mm thick PDMS part), containing an array of 240 square micro-wells (150 × 150 × 180 µm), was used for spheroid formation and subsequent culture; a top layer (~5 mm thick PDMS part) containing a network of microchannels and accessible via open wells, was designed for cell injection, medium perfusion and drug gradient formation. In the top layer, two side channels (35 µm depth) were connected to a central channel (containing the micro-wells) by an array of smaller microchannels (7 µm depth) (Fig. 1C). First, a cell suspension was injected in one of the central wells (W_3–4_ in Fig. 1A), creating a flow along the central channel that allowed cells to sediment into the micro-wells, as previously reported^32,33^. Due to the non-adherent conditions of the micro-wells, single cells formed a compact multicellular spheroid within 2 days of culture (Video S1 in SI). The seeding protocol resulted in a decreasing number of cells seeded in each column of the micro-well array, creating a range of spheroid sizes (50–150 µm) that could be tested simultaneously (Fig. 1D).

Subsequently, by creating a hydrostatic pressure difference between the external (W_1–2–5–6_ in Fig. 1A) and the central reservoirs (W_3–4_ in Fig. 1A), an exponentially decaying flow was created over the spheroid array in the central channel, which had two purposes. First, it was used to facilitate continuous medium exchange (every 24–48 hours) without applying shear stress to the spheroids, as well as removing waste products. Using this protocol, spheroids were cultured in the devices for up to 21 days when using cell lines, and up to 12 days when culturing biopsy-derived primary cells (Fig. 1E). Second, it could be used to create a stable, long-lasting drug concentration gradient (Fig. 1B) across the spheroid array without the need of external instrumentation. As a result, all the spheroids in the same row were exposed to the same concentration, whilst an almost linear concentration gradient was achieved along the columns of the micro-well array (Fig. 1B,C). The latter feature provided the means for obtaining an 8-point concentration response curve per device, where each point is the mean of the readouts of all the spheroids belonging to the same row and allowing for the required spheroid size to be selected.

### Long-lasting self-generated compound concentration gradients

To generate a chemical concentration gradient in the central channel, a near steady-state flow must be maintained, compensating for molecular diffusion across the spheroid array. In the absence of external equipment, this was achieved by carefully designing the resistive and capacitive microfluidic network and by creating overflow ports in both reservoirs connected to the central channel (W_3–4_ in Fig. 1A). To create the concentration gradient, a volume of a drug solution was pipetted into the reservoirs of a side channel (W_1–2_ in Fig. 1A) and the same volume of complete medium was pipetted into the reservoirs of the other side channel (W_5–6_ in Fig. 1A), whilst both reservoirs of the central channel contained a smaller volume of complete medium. This created a symmetrical hydrostatic pressure-driven flow across the micro-well array that simultaneously transported drug solution and medium from each side channel, respectively, towards the reservoirs of the central channel. The hydrostatic pressure difference, *ΔP* = (*P*(*C*_*S*_) *− P*(*C*_*C*_)), between the side reservoirs and central reservoirs of the microfluidic network followed an exponential decay over time (Fig. 2). This was primarily dependent on the fluid height (*h*) in each reservoir (hydrostatic pressure being *P* = *ρgh*, *ρ* is the fluid density and *g* the gravitational constant) and determined the magnitude of the flow rate in the central channel (*ΔP* = *RQ*, where *R* is the fluidic resistance of the channel network and *Q* the volumetric flow rate). In the absence of overflow ports (Fig. 2A), the liquid level between inlet and outlet would eventually equilibrate due to a decreasing *P*(*C*_*S*_) and an increasing *P*(*C*_*C*_). Conversely, when overflow ports were created in the central reservoirs (Fig. 2B), *P*(*C*_*C*_) remained constant after the fluid volume had reached the height of the overflow port, whilst the liquid level in the side reservoirs decreased to the fluid level of the central reservoirs. Consequently, the rate of change of *ΔP* decreased considerably faster in the absence of an overflow, leading to a quick decrease of the flow rate in the central channel, which prevented a concentration gradient to be maintained for longer time periods. Therefore, the introduction of overflow ports and an appropriate microfluidic design allowed fine-tuning of the flow in the central channel to compensate for molecular diffusion, thus achieving a long-lasting concentration gradient (Fig. 2B).

In order to estimate the volumetric flow rates within the microchannel and chamber network, an analogous electrical circuit of the microfluidic network was created^27^, where reservoirs were considered as electrical capacitors and the microchannel network as an electrical resistive mesh. This circuit was simulated in PSpice (Fig. S1 in SI) to estimate the hydrostatic pressure patterns in each reservoir and a simplified analytical model (Fig. S2 in SI) was derived, leading to the following set of equations:

Overflow case:

3a $$P({C}_{S})=\rho g({h}_{{s}_{in}}-({h}_{{S}_{in}}-{h}_{{C}_{in}})(1-{e}^{-\frac{t}{{R}_{T}4{C}_{S}}}));\,P({C}_{C})=\rho g({h}_{{C}_{in}});$$

No overflow case:

3b $$\begin{array}{rcl}P({C}_{S}) & = & \rho g({h}_{{s}_{in}}-\frac{({h}_{{S}_{in}}-{h}_{{C}_{in}})}{1+\frac{2{A}_{S}}{{A}_{C}}}(1-{e}^{-\frac{t(1+\frac{2{A}_{S}}{{A}_{C}})}{{R}_{T}4{C}_{S}}}));\\ P({C}_{C}) & = & \rho g({h}_{{C}_{in}}+\frac{2{A}_{S}}{2{A}_{S}+{A}_{C}}({h}_{{S}_{in}}-{h}_{{C}_{in}})(1-{e}^{-\frac{t(1+\frac{2{A}_{S}}{{A}_{C}})}{{R}_{T}4{C}_{S}}}));\end{array}$$

where *P*(*C*_*S*_) and *P*(*C*_*C*_) are the respective hydrostatic pressure values as a function of time; ${h}_{{s}_{in}}$ and ${h}_{{C}_{in}}$ are the initial fluid height values in the side and central well reservoirs, respectively; *R*_*T*_ is the device fluidic resistive network (Fig. S2 in SI) and *A*_*S*_ and *A*_*C*_ are the area of circular side and central well reservoirs, respectively.

Finally, a finite element method (FEM) numerical model (Comsol Multiphysics 3.5) was used to estimate the convective and diffusive behaviour of the compound of choice. To validate the models developed, calcein (100 µM) was used to experimentally visualise the formation and duration of the microfluidic gradient using time-lapse epifluorescence microscopy. The results (Fig. 2C) were compared to the numerical simulation (Fig. S2C,D in SI), demonstrating the suitability of this approach and the robustness of the numerical model. Subsequently, diffusion coefficients for each compound used were estimated or obtained from the literature and simulations were run to identify the appropriate protocols for each compound. Prior to biopsy analysis, experimental validation of the platform was performed using cell lines (Fig. 2D).

### Cancer drug screening assay validation

Cisplatin, a chemotherapeutic agent used in the clinic against a broad range of solid tumours^13,34^, was used on UVW spheroids for the validation of the microfluidic drug screening assay. UVW spheroids were formed as detailed above and cultured for 5 days prior to the formation of a cisplatin concentration gradient (29–184 µM) in the device (performed in triplicates). In contrast to microfluidic systems operated using external equipment^23,24^, such as syringe pumps, cells seeded with a hydrostatic pressure-driven flow resulted in a progressively decreasing number of cells entering the micro-wells that were further away from the inlet reservoir. Further, since cells are seeded from both sides of the cell culture channel (see W_3_ & W_4_, Fig. 1A) this cumulative effect is seen on both ends of the microwell array. This led to the formation of spheroids with a range of sizes (>90% within the range 50–100 µm in diameter, see Fig. S4B in SI), an outcome that was exploited to assess spheroid size-dependent drug effects. It is worth noting, that whilst a range of spheroid sizes were generated in each row, the variation between rows remained small (Fig. S4A in SI). An almost even distribution of cells in microwells could be achieved by utilising syringe pumps^23^, which can provide a constant velocity.

Drug incubation was maintained for at least 12 hours, whilst devices were kept in an incubator. Drug concentration values were estimated using the numerical simulations for each row of the gradient device. Subsequently, the drug was removed from the device in order to avoid cross-contamination over the spheroid array. Finally, on day 8, spheroid viability was assessed by staining using PI and FDA and the experiments were terminated. Both brightfield and fluorescence images of the spheroids were processed to calculate the temporal evolution of the shape factor (equation (1), Fig. 3A), as well as the viable fraction (equation (2)) on day 8 (Fig. 3B). Viability dyes were only used as end-point measurements due to their potential cytotoxic effects, whereas the additional readout of the shape factor enabled continuous assessment of spheroid health (extracted from daily brightfield images), revealing both short- and long-term drug effects. The shape factor increased after cisplatin incubation in a concentration-dependent manner, with statistically significant increases (p < 0.001) occurring at concentrations ≥ 93.2 µM (Fig. 3A). Concentrations of cisplatin ≥ 159.8 µM produced a significant increase (p < 0.001) in the shape factor 24 hours after drug application, whilst for concentrations (93.2–124.3 µM), the shape factor only increased significantly 3 days post drug application.

Two spheroid groups were arbitrarily created, based on spheroid diameter (Group 1 = 0–75 µm, Group 2 = 76–150 µm) (Fig. 3B), to investigate size-dependent drug effects. EC_50_ values, defined as the concentration of a drug that produces half of its maximum response, were calculated from both brightfield and fluorescent images. Parallel experiments were carried out in triplicates, using just the central chamber of the device, with each device containing a single known drug concentration (0, 29.4, 71.5, 101.2, 155.5, 183.7 µM, respectively) to validate the results obtained from the gradient-generating device. IC_50_ values obtained from these experiments matched results from the gradient device experiments (EC_50_ Fixed concentration = 103.1 µM, EC_50_ Gradient device = 103.7 µM, Fig. S4 in SI), confirming the robustness of the numerical model and experimental protocols developed. Additionally, experiments were performed using the prostate cancer cell line, LNCaP, which was exposed to a concentration gradient of docetaxel (Fig. 3C,D). For both UVW and LNCaP cells, results confirmed the successful formation of a concentration gradient over the spheroid array. The viable fraction parameter proved to be a robust readout for both assays. However, in the case of LNCaP spheroids, the administration of docetaxel resulted in a reduction of spheroid size with increasing concentration, rather than increased spheroid disaggregation. Consequently, the shape factor did not vary significantly as was observed in the case of UVW spheroids and cisplatin. To further investigate the use of the shape factor as a readout for spheroid health, correlation analysis was conducted using the Pearson correlation coefficient as described by Thakuri *et al*.^35^ to quantify the correlation between the shape factor and the viable fraction. We observed a strong negative correlation between the viable fraction and the spheroid shape factor, with a correlation coefficient of −0.9333 in the case of the UVW spheroids (Fig. 3C,D). This suggests that, as the viable fraction of a spheroid decreases, its shape factor will increase in a highly correlative manner. This corresponds with our observations and the images shown in Fig. 3D. However, in the case of LnCAP cells shown in Fig. 3C, the correlation coefficient did not suggest a correlation, since these spheroids decreased in size after drug exposure, but remained structurally intact.

### Prostate tumour biopsy screening

**Figure 4:**
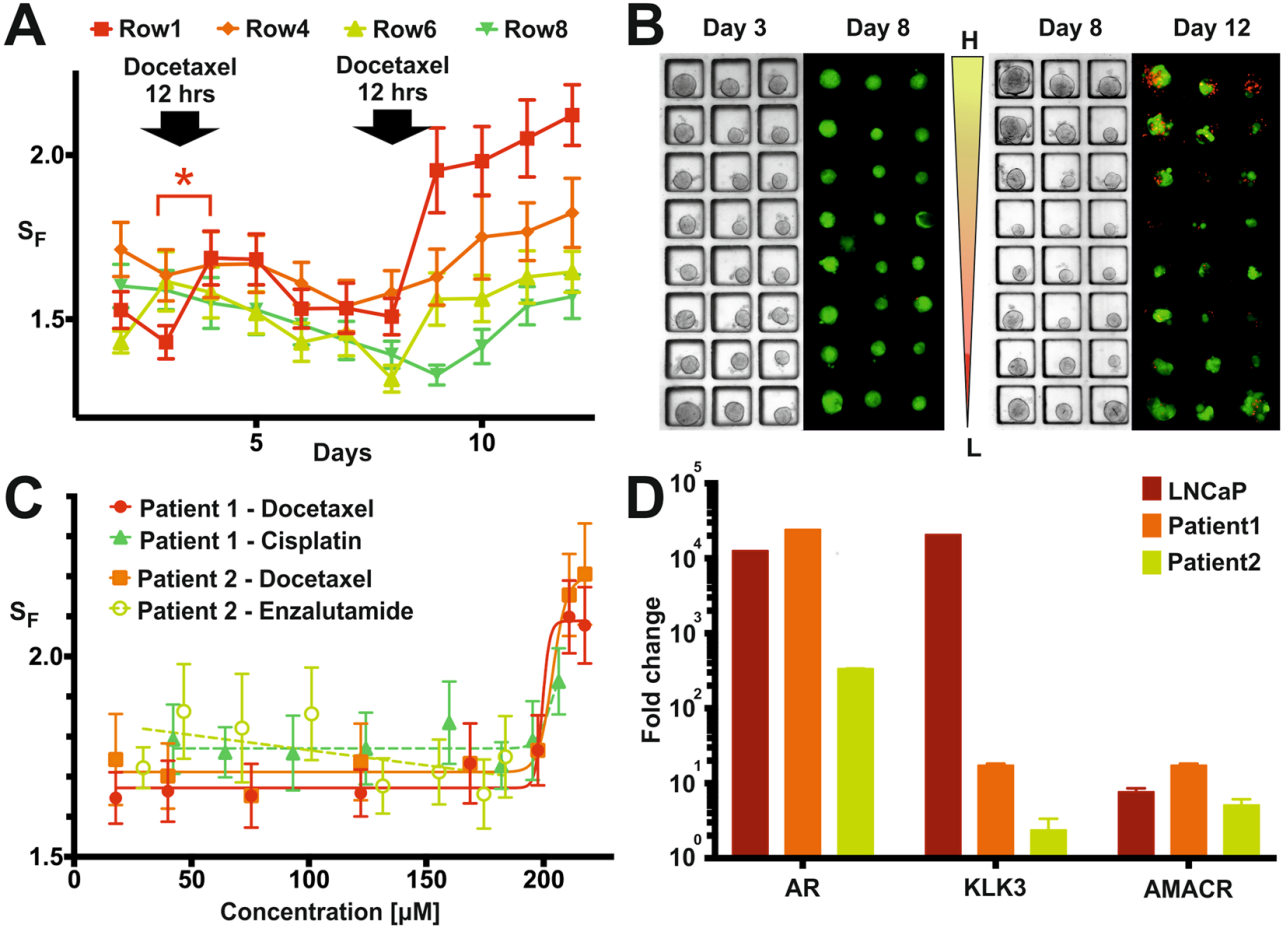
Primary prostate biopsy experiment results. (**A**) Shape factor temporal evolution obtained averaging all the spheroid responses in the highest (Row1), middle (Row4&6) and lowest (Row8) concentration of docetaxel in the case of repeated drug application (only four rows were plotted for clarity). *Represent p < 0.05. (**B**) Representative images from fractionated chemotherapy experiments. From left to right: bright-field (day 3, prior to the first drug incubation) and fluorescent (day 8, experiment terminated) images of biopsy derived spheroids from Patient 1 from one set of 11 devices. No detrimental effect was apparent on day 8; bright-field (day 8 prior to the second drug incubation) and fluorescent (day 12, experiment terminated) images of biopsy derived spheroids from Patient 1 from a second set of 11 devices. A concentration-dependent detrimental effect due to docetaxel application was apparent on day 12. (**C**) Comparison between concentration response curves obtained from Patient 1 and Patient 2 derived spheroids using the shape factor for a selection of drugs tested. Only docetaxel produced a significant increase in cell death for both patients at the highest concentrations. (**D**) RT q-PCR validation of the presence of prostate cancer cells within the cultured cells, using expression of AR, KLK3 and AMACR. PNT2 cells served as reference group and ActinB as a housekeeping gene. Values are fold change ± standard error.

Primary prostate cancer tissue from two different patient biopsies was cultured for two weeks prior to microfluidic experiments. Morphological assessment of the expanded biopsies was conducted to assess the presence of multiple cell types in each culture. From each biopsy preparation, a cancer-cell enriched single-cell suspension was used to prepare a number of gradient-generating devices. Depending on the tissue proliferation during expansion, between 13 and 22 devices could be seeded per biopsy. Alongside the microfluidic experiments, RT-qPCR was performed for each biopsy to assess the presence of prostate cancer cells and several prostate cancer biomarkers: androgen receptors (ARs), prostate-specific androgen (KLK3) and alpha-methyl-acyl-CoA racemase (AMACR). Expression of these markers was quantified using a benign prostate cell line (PNT2) as a reference and a prostate cancer cell line (LNCaP, Fig. 4D) for comparison. Pathology reports confirmed that both patients in the study had prostate cancer. Spheroids were allowed to form in the microfluidic device for 3 days in all experiments, before each condition was tested in triplicates. To demonstrate the capabilities of the microfluidic platform, different proof-of-concept experiments were carried out for each biopsy.

Using cells derived from Patient 1, fractionated chemotherapy was mimicked, by applying a number of drugs (enzalutamide, docetaxel or cisplatin), each drug being a separate experiment. On day 3 of culture, a drug gradient (8.9–108.8 µM docetaxel, 14–92 µM enzalutamide or 21.1–103.2 µM cisplatin) was applied to the spheroids for 12 hours. After drug removal, spheroids were monitored daily using brightfield microscopy until day 8. In a number of devices (a mixture of devices either with 8 rows of 150 um well or 5 rows of 250 um wells) spheroid viability was determined on day 8 by staining with PI and FDA (Fig. 4B). In the remaining devices, a second drug gradient was reapplied for 12 hours (for all conditions tested), doubling the concentration ranges (17.8–217.6 µM docetaxel, 29.4–183.7 µM enzalutamide and 42.2–206.5 µM cisplatin). Monitoring continued until day 12, when viability was measured using FDA and PI (Fig. 4B). Following the first drug application, viability staining showed 100% viability across the entire concentration range for all drugs (Fig. S5A). However, for docetaxel, a transient increase (p = 0.049) in shape factor was observed on day 4 for the highest concentration applied (108.8 µM, Fig. 4A, Row 1). Following the second drug application, docetaxel produced an increase (p < 0.001) in shape factor for concentrations ≥ 122.1 µM, with viability staining following a similar trend, resulting in an EC_50_Patient 1_ = 161.7 µM. RT-qPCR results indicated elevated expression of all markers in LNCaP cells with respect to PNT2 cells. Further, the increased expression of AR, KLK3 and AMACR in Patient 1 and 2 suggests the presence of prostate cancer cells within the mixed biopsy population.

To confirm the robustness of the protocol, the same higher concentrations of drugs were tested on spheroids derived from the biopsy preparation of Patient 2. Following cell seeding, concentration gradients of several drugs were applied on day 3 of culture (docetaxel, enzalutamide ± DHT enzalutamide in a range 29.4–183.7 µM). On day 8, viability staining was conducted in all devices and experiments were terminated. Out of all conditions tested, only docetaxel produced a detrimental effect to the spheroids, with similar concentration-response curves and EC_50_ values as seen for Patient 1 (EC_50___Patient 1_ = 161.7 µM, EC_50___Patient 2_ = 165.2 µM, Fig. 4B). In order to compare spheroid responses between the two patients, only spheroids with a similar diameter were considered (25–95 µm diameter, Fig. 4C).

## Discussion

Our approach provides a novel microfluidic solution for self-generating drug concentration gradients across hundreds of multicellular spheroids in a shear stress-free manner without the need of external fluid actuation. We have demonstrated that a concentration gradient remained stable for 12–16 hours (Fig. 2C,D). These concentration gradients can be re-established over time, a feature that was utilised here to mimic fractionated chemotherapy and that, alternatively, could be used to create extended, continuous drug incubation in multiples of 12–16 hours with the current device layout.

When the compound of interest cannot be visualised via microscopy (most anticancer agents are non-fluorescent), it is important to assure that the expected gradient pattern has occurred. The validated numerical models developed (Eq. 3) describe the temporal evolution of the fluid volumes in all wells. When compared to the experimental values, they provide a good measure of whether the gradient had developed as expected. Therefore, in all our experiments, once a concentration gradient had been applied for 12 hours, measurement of the remaining liquid volumes in each reservoir provided indication of successful device operation. If the liquid volumes in the reservoirs differed >10% from the expected value, the experiment was discarded. This procedure served to detect whether external factors, which were not included in the simulation, such as the formation of a liquid meniscus in the overflow ports or excessive evaporation in the reservoirs, compromised the device operation. Our 3D FEM simulation estimated that, in the worst-case scenario, ~10% change in the gradient concentration pattern could occur over 24 hours. Further, once the gradient has been established, minor variation in concentration can occur across the wells belonging to the same row (Fig. S3 and Video S2) due to fringe effects, especially in rows 1 and 2. However, this error was negligible (<1%) in the majority of rows according to our experiments with calcein. To account for potential variations, spheroids positioned in the first and last three columns of the array were excluded from the analysis. Overall, due to the external factors identified above, a 13-hour window (1 hour for gradient establishment and 12 hours of drug incubation, Fig. 2C) was selected to prevent unwanted effects. Further, improvement to the fabrication procedures (e.g. by using injection moulding) and replacement of PDMS (e.g. in favour of polystyrene) are expected to greatly diminish the impact of surface treatment, evaporation and liquid absorption into bulk PDMS, thus extending the duration of the chemical gradient.

Once established, the microfluidic gradient generates concentrations spanning two orders of magnitude (e.g. 29.4–183.7 µM, Fig. 4C). With respect to typical drug screening in 2D (spanning both nanomolar and micromolar concentrations), this concentration range is restricted. However, a range of two orders of magnitude is frequently sufficient, since it is well documented that spheroids, derived either from cell lines or primary tissue, are more resistant to chemotherapeutic agents than cell monolayers. Hence, the use of an extended concentration range is not warranted. The concentration values of docetaxel, enzalutamide and cisplatin used in this work are in agreement with previous studies, which also suggested limited drug sensitivity to lower concentrations^8,36,37^. Additionally, when screening patient derived tissue, it becomes a priority to identify which drug or combination of drugs are affecting the spheroids in order to inform on the therapeutic approach most suited. In these conditions, maximising the number of tests performed over the larger number of spheroids (due to the heterogeneity of the tissue) becomes predominant over the identification of an EC50 value. Finally, although the majority of our experiments were performed using a spheroid array comprising 8 rows of square wells of 150 µm size, we also carried out experiments when screening biopsy tissue using arrays comprising 5 rows of square wells of 250 µm size (Fig. S6) to account for screening larger spheroids. With the current design layout for gradient formation, this identified a trade-off between the number of points per curve against the spheroid size which could be an important parameter when studying drug effects. In the future, the cell culture channel could be widened to increase the number of concentration points (i.e. obtaining further rows). However, while one could argue that the lower drug concentration in the array could act as a control line, an independent separate device would have to be used for control experiments.

The ability to culture a range of spheroid sizes within the device allows us to detect potential size-dependent drug effects. For example, Fig. 3B shows a size dependent response from UVW spheroids treated with cisplatin, where both shape factor and viable fraction vary depending on spheroid diameter. EC_50_ values obtained from both readouts indicate that larger spheroids were more sensitive to cisplatin than smaller ones. This deserves further investigation but could be related to the presence of hypoxic cells in larger spheroids, which has been shown to increase the effectiveness of cisplatin in some cell lines^38^.

The heterogeneous nature of the tissue from a tumour biopsy, whilst ultimately an advantage for representing the real tumour microenvironment, provides challenges in terms of the different cell types contained (e.g. prostate cells, fibroblasts and cancer associated fibroblasts) and the overall amounts of cancer cells. The media employed in the current experiments aimed to preferentially select for and promote epithelial cancer cell expansion through the addition of multiple additives including Fibroblast Growth Factor 10 (FGF10) and reduce fibroblast growth using Cholera Toxin^39,40^. However, a small percentage of fibroblasts remained. In order to characterise the cell population, RT-qPCR was performed. Results demonstrated that primary cultures expressed both AR and prostate-specific androgen (PSA), suggesting that the AR was functional (Fig. 4D). However, despite the presence of seemingly functional AR, no concentration of enzalutamide (29–184 µM), a nonsteroidal AR-antagonist and signalling inhibitor, tested in this study for both Patient 1 and 2, had any effect on viability or proliferation. This is similar to the observations made by Gao *et al*.^41^, who showed that the majority of organoids tested in their study were resistant to enzalutamide, and could be for a number of reasons. Firstly, it has been shown that mutations, resulting in structural changes in the AR, can mediate enzalutamide-resistance^42,43^. The majority of patients will eventually develop resistance to enzalutamide, in addition to significant occurrence of *de novo* resistance^44,45^. Secondly, the presence of fibroblasts in the spheroids might confer some degree of chemotherapy-protection, which is a well-documented occurrence^46,47^ and can appear in a broad range of cancers, including enzalutamide-resistance in prostate cancer^48^. Fibroblast-mediated resistance is one mechanism that could explain the high extent of resistance shown by the primary prostate spheroids to all drugs tested in this project. Several publications^41,49^ have shown the large range of possible drug responses that can be obtained from organoids, not only within the same cancer type, but even within the same patient. However, since only two patients were considered in this study, no larger assumptions can be made.

In addition to the advantages obtained from miniaturised microfluidic assays, the ability to use label-free readouts, such as the shape factor, is an extremely valuable tool to inform on drug effects. Spheroid diameter is a commonly used parameter to establish drug efficacy, but is not always reliable when spheroid integrity is compromised^50^, which can occur after drug incubation or insufficient culture conditions. Our study has shown that the combination of a spheroid-based assay with image analysis of bright-field images, can provide a sensitive measure of spheroid response to a drug, as previously reported^13,30,31,51–53^. Since the shape factor is a non-destructive measure of spheroid integrity (compared to dye-based single end-point measurements), it can provide valuable additional insights into the evolution of spheroid health over time, both for drug and control experiments. If dyes were used for daily assessments, it would require at least one gradient device per condition to be terminated every day, limiting the number of possible screens. This is demonstrated in Fig. 4A, where the application of 108.8 µM docetaxel (Row 1) caused a transient change in shape factor around day 4. In this particular case, if viability staining were the only readout available, no detrimental effect could have been detected on day 8. Further, as shown in Fig. 3A, spheroid disaggregation increased in all treatment groups for 3 days after treatment, which highlights the importance of identifying a suitable day for end-point analysis. However, it is worth noting that concentration-response curves generated from the shape factor and viable fraction (Fig. 3C) did not always match. This may be due to the cancer model used and the drug’s mechanism of action. Importantly, the ability to provide multiple readouts from continuous monitoring and image analysis strongly mitigates the risks of misinterpreting drug effects.

## Conclusion

There is an ongoing need to develop more predictive and rapid means of profiling cancer patients to allow stratified and personalised medicine solutions. Current investigation of anticancer therapeutic relies heavily on animal models (involving procedures implanting cancerous tissue from a human tumour into an immune-deficient or genetically engineered mouse). These approaches are very resource-, time- and cost-intensive. Our microfluidic technology offers a new solution for extensive anticancer compound screening using 3D micro-tumour models generated from cancer patient biopsies. For this, we anticipate applications in the screening of tissue that is notoriously difficult to analyse due to very small size biopsies (e.g. oesophageal tumour biopsies and other tissues obtained by fine-needle aspiration). Remarkably, whilst combination therapy is an emerging treatment approach for many cancer types, *in vitro* combinatorial screening of patient-derived 3D spheroids remains challenging due to the large cell number required. Therefore, it is conceivable that our system and protocols, when applied to combination chemo- and radio-therapy, may offer new and cost-effective avenues to future personalised medicine solutions.

## Electronic supplementary material

Supplementary Information

Spheroid formation

Gradient formation

## Data Availability

The datasets generated during and/or analysed during the current study are available from the corresponding author on reasonable request.
