# Supplementary material for: Drug screening of biopsy-derived spheroids using a self-generated microfluidic concentration gradient
Source: Sci Rep. 2018 Oct 2;8:14672. doi: 10.1038/s41598-018-33055-0 (PMC6168499; doi:10.1038/s41598-018-33055-0)
Supplement: Supplementary file 1 — Supplementary Information [file 41598_2018_33055_MOESM1_ESM.docx]

**Supplementary Information (SI)**

**Drug screening of biopsy-derived spheroids using a self-generated microfluidic concentration gradient**

Theresa Mulholland^1^, Milly McAllister^2^, Samantha Patek^2^, David Flint^3^, Mark Underwood^4^, Alexander Sim^5^, Joanne Edwards^2^ and Michele Zagnoni^1*^

*^1^ Centre for Microsystems and Photonics, Electronic and Electrical Engineering, University of Strathclyde, Glasgow, G1 1XW, UK*

*^2^ Institute of Cancer Science, College of Medical, Veterinary and Life Sciences, University of Glasgow*

*^3^ Strathclyde Institute of Pharmacy and Biomedical Sciences, University of Strathclyde, Glasgow, G4 0RE, UK*

*^4^ Department of Urology, Queen Elizabeth University Hospital, Glasgow, G51 4TF, UK*

*^5^ AMS Biotechnology (Europe) Ltd, Abingdon, UK*

** Corresponding author: michele.zagnoni@strath.ac.uk*

**Video S1**………………………………….………………..page2

**Video S2**………………………………….……………..…page2

**Table S1**………………………………….………………..page2

**Figure S1**……………………………….…………………page3

**Figure S2**……………………………….…………………page3

**Figure S3**……………………………….…………………page4

**Figure S4**…………………………….……………………page5

**Figure S5**……………………………….…………………page6

**Figure S6**……………………………….…………………page6

**Figure S7**……………………………….…………………page7

**RNA extraction details**…………………………….……page7

**cDNA synthesis details**…………………………………page8

***Video S1.*** Time-lapse recording (12 frames/hour) of spheroid formation within the micro-wells of a microfluidic device from a seeded cell suspension of UW cells over 24 hours.

***Video S2.*** Time-lapse recording (12 frames/hour) of calcein gradient formation within the micro-wells of a microfluidic device over 16 hours.

***Table S1.*** List of additives used in advanced DMEM/F12 (Invitrogen, UK) for human primary prostate cell culture medium.

| **Supplement** | **Manufacturer** | **Concentration** |
| --- | --- | --- |
| EGF | Sigma Aldrich | 1 μM |
| N-acetyl-cysteine-L | Sigma Aldrich | 1.25 mM |
| Human R-spondin-1 | PeproTech | 10 ng/ml |
| Human Noggin | PeproTech | 10 ng/ml |
| DHT | Sigma Aldrich | 1 nM |
| FGF10 | BioVision | 1 ng/ml |
| FGF2 | BioVision | 0.1 ng/ml |
| SB202190 | Sigma Aldrich | 10 μM |
| Y27632 | USBiological Life Sciences | 10 μM |
| Cholera Toxin | Sigma Aldrich | 1 μg/ml |
| Amphotericin B | Invitrogen, UK | 2.5 µg/ml |
| Penicillin/Streptomycin | Invitrogen, UK | 50 U/ml, 50 μg/ml |
| L-glutamine | Invitrogen, UK | 2 mM |
| B-27 | Invitrogen, UK | 20 ml/l |


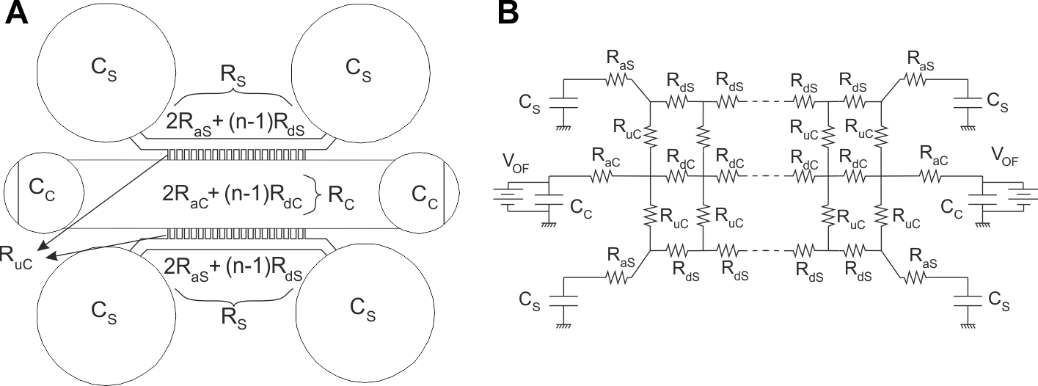


**Figure S1.** Considering the geometrical features of the microfluidic device developed (A), an analogue electrical circuit (B) was created to determine the pressure pattern in each reservoir using a numerical simulation in PSpice, where the initial fluid level in each reservoir was modelled as an initial electrical charge in each capacitor. C_S_ and C_C_ indicate the capacitance of the side and central reservoirs. R_S_ and R_C_ represent the resistance of the side and culture channels, obtained as the sum of the distributed resistances of each channel. R_uC_ indicate the resistance of each microchannel.


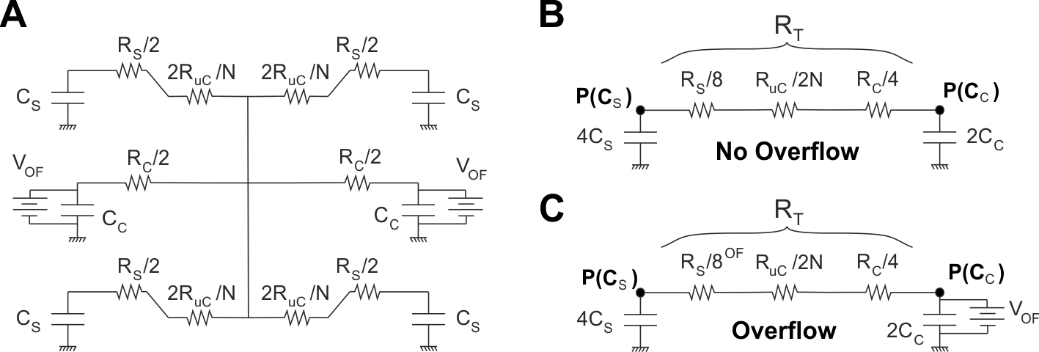


**Figure S2.** A simplified analogue electrical circuit (A) was derived from that of figure S1 to extract an analytical expression of the pressure patterns in each open well reservoir (P(C_S_) and P(C_C_)) that matched those obtained from the numerical simulation. The circuit could be further simplified to model the flow from the side reservoirs to the central reservoirs in the presence (C) and absence (B) of overflow ports created in the central reservoir. Overflow ports were modelled as a constant potential generator connected to the central reservoir capacitors.


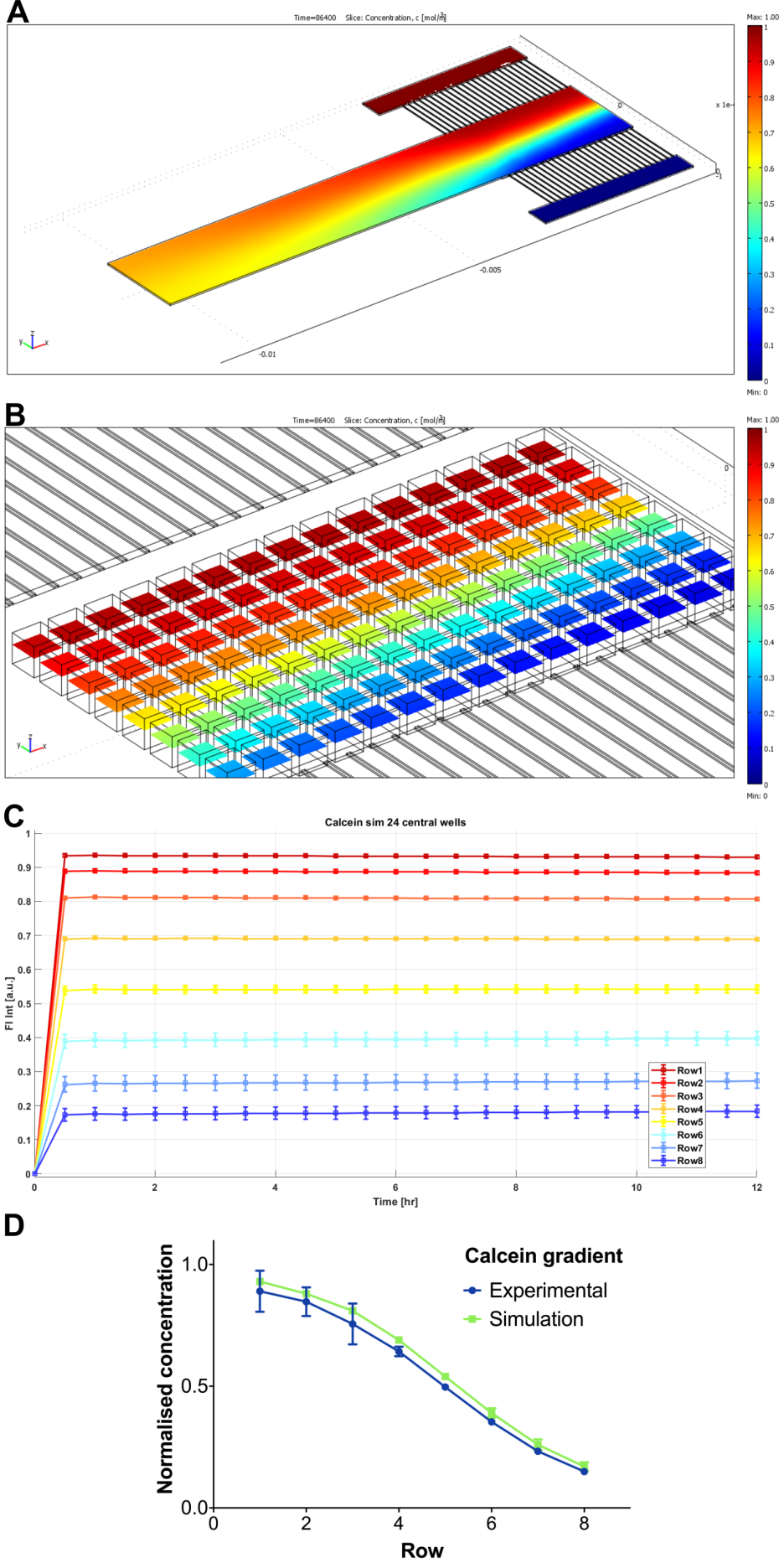


**Figure S3.** 3D numerical model of the microfluidic device developed. A) Representative plot of calcein distribution in the central channel during gradient formation. B) Representative plot of calcein distribution in the micro-wells during gradient formation. C) Plot of the average value (normalised ± standard error) of calcein in each row of the spheroid array. D) Comparison between experimental and simulated calcein gradient. Average values from all wells in each row indicate calcein concentration and is represented as normalised values to max (100 uM) + standard deviation.


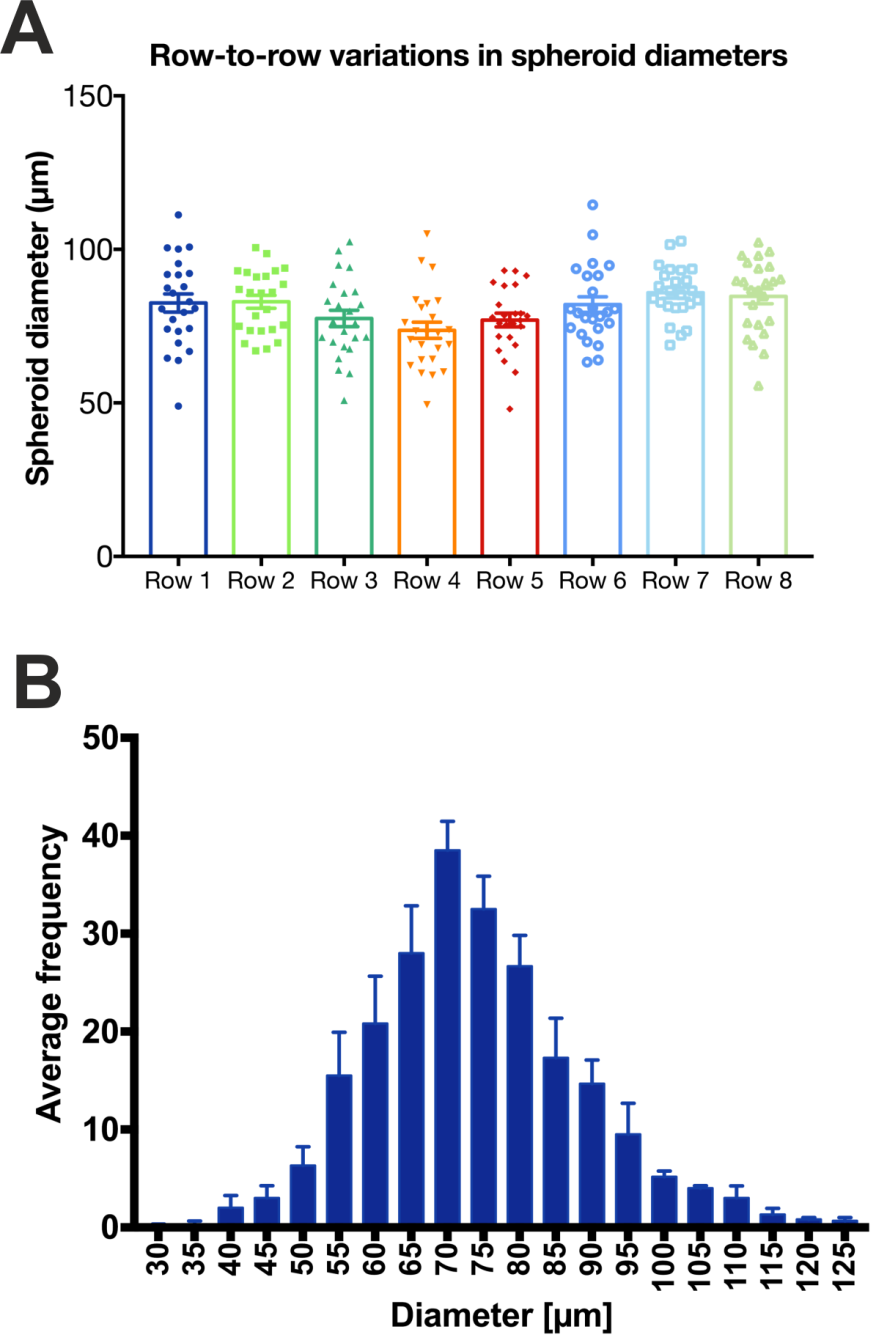


**Figure S4.** A) Comparison between spheroid size in each row of a representative gradient device, showing mean + standard error of the values, as well as individual distribution of points. B) Histogram plot of the size distribution of spheroids from 6 different devices (error bars represent standard error).


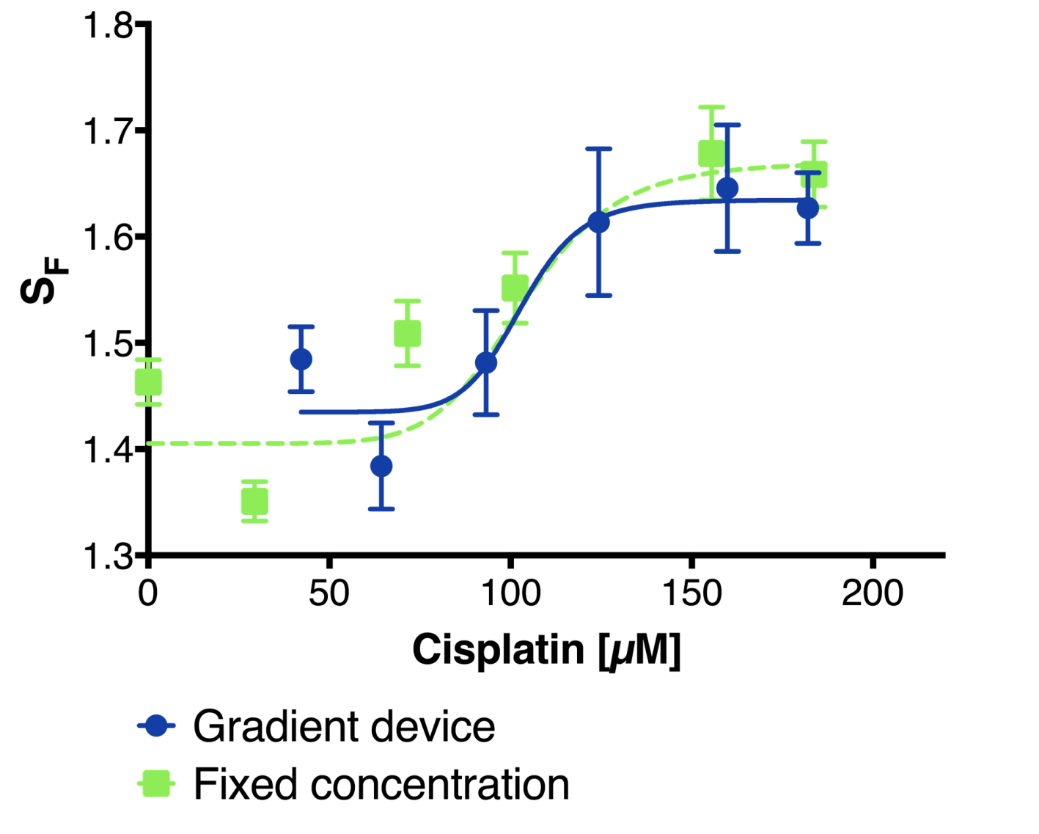


**Figure S5.** Comparison between concentration response curves obtained using the shape factor between a gradient generating device and fixed concentrations, showing similar EC_50_ values. EC_50_ (Fixed concentration) = 103.1 µM, EC_50_ (Gradient device) = 103.7 µM.


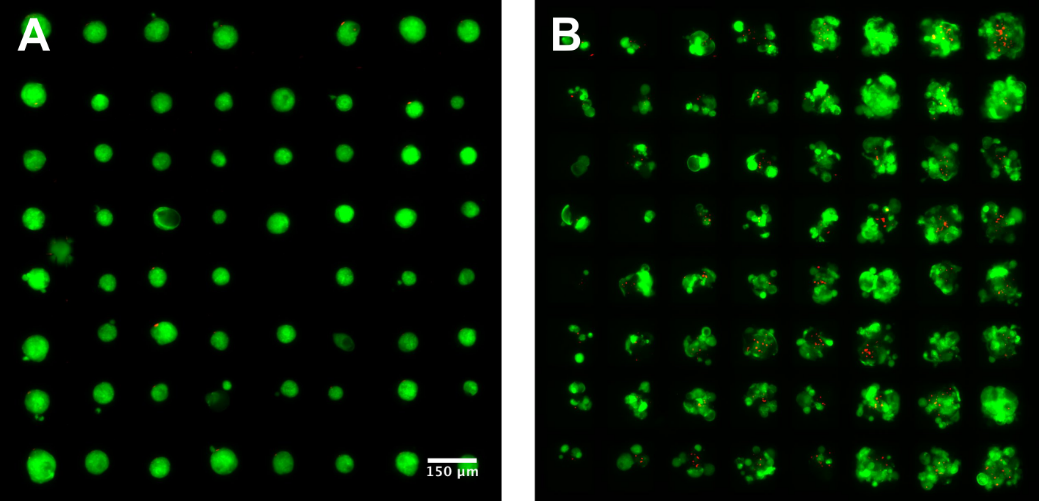


**Figure S6**. Primary prostate biopsy-derived spheroids generated from Patient1. A) After 5 days of culture post application of a docetaxel concentration gradient (8.9 - 108.8 µM) over the spheroid array, no sign of detrimental effects was identified when assessing viability using FDA (green) and PI (red) staining. B) Representative image of vehicle control (2.2% DMSO) culture (no drug application) after 12 days in the microfluidic device. Over time, biopsy derived spheroid present morphological changes with respect to earlier days (up to day 8), but retain a high viable condition. Control experiments in medium did not show any difference with respect to vehicle controls.


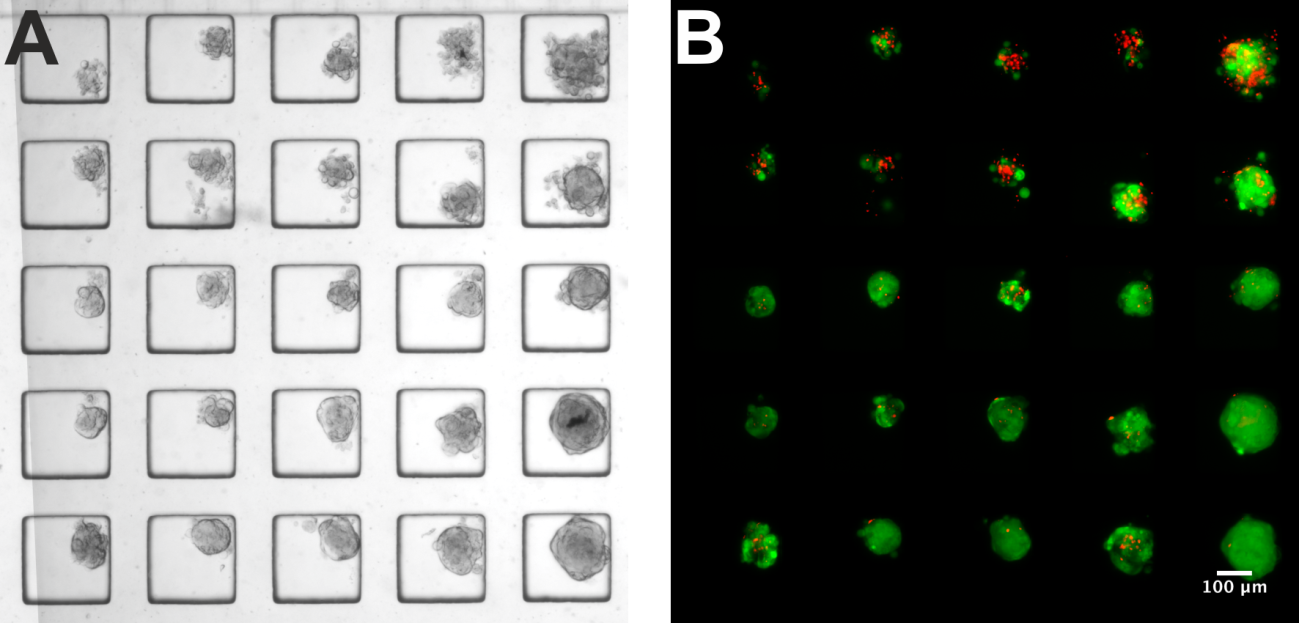


**Figure S7**. Primary prostate biopsy-derived spheroids generated from Patient2. A & B) Brightfield and fluorescent images obtained post application of a docetaxel concentration gradient (17.5 – 217.6 µM) using square wells of 250 µm size, showing similar effects to what has been obtained with devices comprising square wells of 150 um size. Viability stain was done using FDA (green) and PI (red).

**RNA extraction**

RNA was extracted from the primary prostate cell cultures described above using the RNeasy Mini kit (Qiagen, Manchester, UK). Cells were cultured in appropriate growth medium in a T75 flask to approximately 70% confluency. Cells were trypsinised and neutralised with appropriate growth media. Cells were then transferred to a corning tube, and centrifuged at 1200 rpm for 3 minutes. The supernatant was aspirated. Cells were re-suspended in 1 ml of PBS and transferred to a nuclease free tube. Cells were centrifuged again at 1200rpm for 3 minutes and supernatant aspirated. To allow all RNA to be made available, cell membranes were disrupted and the cells homogenised. Cells were lysed using 350 μl of RLT lysis buffer. Cells were homogenised by repeatedly aspirating the cell suspension through a 26G needle and 1 ml syringe. 350 μl of 70% ethanol was added before immediately transferring the homogenised cell sample to a spin column and centrifuged for 15 seconds at 8000 x g. Flow through was discarded. Then, the cell sample was washed in RW1 buffer to remove large biomolecules that were not attached to the spin column membrane. RW1(700 μl) was applied to the spin column membrane and centrifuged for 15 seconds at 8000 x g. Flow through was discarded. The cell sample was washed again with a mild washing buffer (RPE buffer) that removes any residual salts from the RNA extraction process. Two washes with 500 μl RPE buffer were performed, centrifuging for 15 seconds at 8000 x g after the first wash and two minutes at 8000 x g. Flow through was discarded after each wash. Finally, the spin column was centrifuged for one minute at 8000 x g to dry the membrane. The spin column was placed into a new collecting tube. RNase free water (30 μl) was added directly to the spin column and centrifuged for one minute at 8000 x g to elute RNA. The spin column was discarded and the RNA was stored at -80°C until use. DNA was removed from the sample prior to cDNA synthesis to avoid residual DNA to be amplified in quantitative real time-PCR (RT-qPCR). Firstly, the quantity and purity of the RNA before the DNA extraction step was quantified using the Nanodrop. To 2 μg RNA, 2 μl 10x DNase I reaction buffer (Promega, Southampton, UK), 1ul DNase I (Promega, Southampton, UK), 1ul RNase OUT (Life Technologies, Paisley, UK) and nuclease free water to a volume of 20ul was added. The mixture was incubated at room temperature for 15 minutes, before inactivating the DNase solution by the addition of 1μl of Stop solution (Promega, Southampton, UK). The solution was then heated to 65°C for ten minutes. After heating, the solution was stored on ice and transferred to the Nanodrop to determine the quantity and purity of the RNA post-DNA elimination.

**cDNA synthesis**

For a 100 μl cDNA synthesis reaction, 1 μg RNA from the cell line of interest was added to 5 μl random primers (Life Technologies, UK) and topped up with nuclease-free water to a volume of 62 μl. The RNA solution was heated to 65°C for ten minutes. After heating, 20 μl of 5x FS buffer,2.5 μl of RNase OUT, 2.5 μl of Superscript II reverse transcriptase, 10 μl of 10 nM dNTP and 3.5 μl of DMSO was added to the RNA solution to make a final volume of 100 μl. The RNA solution was heated for 10 minutes at 25°C, then 30 minutes at 50°C before heating to 85°C for 5 minutes to inactivate the transcriptor. cDNA was stored at -20°C until use.
